# Topologically optimized magnetic lens for magnetic resonance applications

**DOI:** 10.5194/mr-1-225-2020

**Published:** 2020-10-12

**Authors:** Sagar Wadhwa, Mazin Jouda, Yongbo Deng, Omar Nassar, Dario Mager, Jan G. Korvink

**Affiliations:** 1 Institute of Microstructure Technology, Karlsruhe Institute of Technology, Hermann-von-Helmholtz-Platz 1, 76344 Eggenstein-Leopoldshafen, Germany; 2 State Key Laboratory of Applied Optics (SKLAO), Changchun Institute of Optics, Fine Mechanics and Physics (CIOMP), Chinese Academy of Sciences, Dongnanhu Road 3888, Changchun 130033, China

## Abstract

Improvements to the signal-to-noise ratio of magnetic resonance detection lead to a strong reduction in measurement time, yet as a sole optimization goal for resonator design, it would be an oversimplification of the problem at hand. Multiple constraints, for example for field homogeneity and sample shape, suggest the use of numerical optimization to obtain resonator designs that deliver the intended improvement. Here we consider the 2D Lenz lens to be a sufficiently broadband flux transforming interposer between the sample and a radiofrequency (RF) circuit and to be a flexible and easily manufacturable device family with which to mediate different design requirements. We report on a method to apply topology optimization to determine the optimal layout of a Lenz lens and demonstrate realizations for both low- (45 MHz) and high-frequency (500 MHz) nuclear magnetic resonance.

## 1 Introduction

### 1.1 Signal-to-noise ratio (SNR) in magnetic resonance (MR)

Nuclear MR spectroscopy and imaging are powerful tools for determining the molecular structure of chemical substances or for studying the anatomy of organisms. For MR measurements, it is important to achieve a high SNR to obtain a high-resolution spectrum or a highly resolved image, which also leads to a reduction in the overall measurement time. The relationship between the SNR and the magnetic field produced by the coil was derived by [Bibr bib1.bibx11] and can be reduced to

1 $$\text{SNR}\propto \frac{VB_{1}}{I\sqrt{R}},$$

where $B_{1}$ (in T ) is the radiofrequency magnetic field produced by the coil in a direction normal to the polarizing field $B_{0}$ , V (in $m^{3}$ ) is the sample volume, I (in A ) is the current flowing through the coil, and R (in ohms ) is its AC resistance. From the SNR equation (Eq. 1) it can be deduced that for a given magnitude of $B_{1}$ and a constant I flowing through the coil, the SNR depends directly on the sample volume; therefore, for a smaller volume of the sample, the SNR degrades significantly. To improve the SNR, it is important to improve the filling factor of the coil, which is the geometrical relation between the sample volume and the size of the useful $B_{1}$ volume of the coil. This can be achieved by increasing the magnetic field penetration through the sample whilst maintaining a constant value of the $B_{1}$ field.

From the statement above, it is self-evident that reducing the size of the coil will improve its filling factor, which leads to the SNR enhancement, since the strength of Faraday induction increases with a reduction in the distance between the coil and the sample. The miniaturization of coils, as for example discussed by [Bibr bib1.bibx14], comes with its additional advantages and limitations. The efficiency of the coil increases as the desired magnetic field can be achieved with lower electrical power, yet the use of relatively bulky capacitors for matching and tuning cannot be avoided, since the electrical length scales inversely with the frequency in Maxwell's equation. To tune and match the coil at the frequency of operation, the electrical connections for the capacitors need to be established close to the coil, which is disadvantageous.

In some cases, the reception coil and its capacitors cannot be placed too close to the sample or specimen, e.g. when performing MRI on small living organisms or for sensitive spectroscopy of small samples. In such scenarios, the improvement of the filling factor is remedied by using a Lenz lens (LL) [Bibr bib1.bibx15] which focuses the magnetic field produced by a larger coil into a smaller sample region. Their working principle simply follows Lenz's law of induction, which augments Faraday's law. The transmitter coil induces a current in the outer loop of the LL and by design forces the induced current to also flow to an inner loop but directed in the opposite sense. The dimension of the inner loop is such that it encircles the sample completely. This simultaneously results in a localized magnetic field amplification within the inner loop and a zeroing of the field in the outer loop. Since an LL is broadband up to its high resonance frequency (usually in the GHz range), these devices can be used over a wide range of frequencies without additional tuning. The field amplification produced by an LL depends on the area ratio of the outer to inner loops. When the LL is limited by the available working space, the total magnification that can be achieved is lowered as was shown by [Bibr bib1.bibx13].

If we focus our attention on the LL, its design needs to be further investigated to improve the amplified field uniformity and to increase the field amplification for cases where the geometrical space for the LL is limited. Although it was shown by [Bibr bib1.bibx13] that tuning and matching the LL at the frequency of operation improved the signal acquired significantly, even for those cases where the design space is constrained, it comes at the cost of losing the broadband nature of the LL and adds the difficulty in maintaining the Q factor of the coil/lens arrangement due to the resonance splitting effect. Since the solution to the problem lies in finding the best possible topology of the metal structure to overcome these issues, it prompted us to formulate a method where the tailoring of the field could be controlled mathematically while searching for the optimal design.

In this paper, we now explore the use of computational optimization to “discover”, via inverse design, a novel distributed metallic track arrangement that produces the same effect as a Lenz lens. The computational procedure will aim to maximize the magnetic field flux (i.e. the lensing effect) in the sample and at the same time aim for a flux distribution that is as constant as possible. As will be shown, these two requirements are in conflict, so a supervisor will have to balance these requirements depending on the application. Furthermore, the design will depart considerably from the Lenz lens topology and may require additional constraints to ensure manufacturability.

### 1.2 Topology optimization

Topology optimization has been used in various fields for inverse material design, such as for acoustics [Bibr bib1.bibx8], mechanical structures [Bibr bib1.bibx3], electromagnetics [Bibr bib1.bibx19], thermodynamics [Bibr bib1.bibx9], fluidics [Bibr bib1.bibx23], and permanent magnetic systems [Bibr bib1.bibx17], to name a few.

In the field of electromagnetics, topology optimization has been explored for applications such as photonic crystals [Bibr bib1.bibx19], dielectric clocks [Bibr bib1.bibx6], beam splitters [Bibr bib1.bibx18], antennas [Bibr bib1.bibx24], surface plasmonics [Bibr bib1.bibx2], and more. In most of these cases, the electrical permittivities and magnetic permeabilities of the material were used as a function of space to obtain a material distribution.

However, in surface plasmonics [Bibr bib1.bibx2] and antenna design [Bibr bib1.bibx24], the conductivity value of the material was used to realize the desired structures. For the excitation of surface plasmons, the frequency of operation was in the THz range, for which the normal component of the electric field on any boundary is negligible, and the metal domain can be truncated by applying a perfectly electric conductor boundary condition (PEC). When considering metals in the radio and microwave range (3 MHz–300 GHz), it can no longer be considered a PEC due to the skin depth effect, for which the current penetrates up to a certain thickness of the metal before it decays completely.

[Bibr bib1.bibx1] introduced a method to implement impedance boundary conditions (IBCs) for the inverse material design of metals in the microwave range which takes the skin depth effect into account. During the post-processing, they used the PEC condition on the metal's boundaries for the verification of the results.

For our problem formulation, we chose the conductivity function as a material property in the domain rather than on the boundary. The conductivity range between which the material property is interpolated was of the order of $10^{7}$ , which is similar to the ratio of the conductivity of copper ( Cu ) and that of free space. Since the free space conductivity was set close to zero rather than exactly zero, it forced some portion of the current to be normal at the boundary of free space before it decays. This imitates the behaviour of an IBC, which is then used to generate the material design. For post-processing, the IBC was imposed on the boundaries of the optimized lens (OL) geometries to measure the actual enhancement of the magnetic field.

Using the methodology presented in Sects. [Sec Ch1.S2], [Sec Ch1.S3], and [Sec Ch1.S4], we optimized the magnetic lens for Larmor frequencies of (i) 45 MHz and (ii) 500 MHz, where it was assumed that the excitation $B_{1}$ field is oscillating along an axis perpendicular to the OL.

The obtained geometries were then characterized, and the simulation results were verified by NMR experiments described in Sect. [Sec Ch1.S5.SS2]. For the 45 MHz OL design, NMR measurements were performed on a 1.05 T preclinical MRI machine (Bruker, ICON). The 500 MHz OL measurements were performed on an 11.7 T vertical wide-bore superconducting NMR spectrometer (Bruker AVANCE III). To verify the enhancement of the magnetic field and improvement in SNR, a series of nutation spectra of a distilled water sample were taken, both with and without an OL.

## 2 Methodology

In this section, we consider the electromagnetic wave equation that governs the behaviour of the device, derive the equations of the material distribution method, and formulate the objective function with constraint equations with which to obtain an optimized geometrical configuration corresponding to a spatial material distribution.

The time-varying magnetic field B(t) and electric field E(t) can be defined in terms of a time-varying magnetic vector A(t) and an electric scalar potential ϕ(t) . Assuming a time-harmonic behaviour proportional to $e^{j\omega t}$ , where *j* represents the imaginary unit ( $\sqrt{-1}$ ), ω represents the angular frequency and t the time. B and E in the frequency domain are defined as

2B=∇×A,3E=-∇ϕ-jωA.

Here $\nabla \overset{\text{def}}{=}\left(\partial /\partial x,\partial /\partial y,\partial /\partial z\right)$ represents the gradient operator in a Cartesian coordinate frame of reference. By substituting A and ϕ from Eqs. (2) and (3) into Maxwell's equations and simplifying the result by fixing the Lorenz gauge in order to obtain a unique solution, the modified wave equation with the divergence-free condition becomes

4∇2A-ω2μrμ0ϵrϵ0A=-μrμ0σ∇(ϕ)inΩ,5∇⋅∇(ϕ)=0inΩ.

Here $\mu_{r}$ and $\epsilon_{r}$ represent the relative permeability and permittivity of the propagation medium. $\mu_{0}$ and $\epsilon_{0}$ represent the free space permeability and permittivity. σ denotes the conductivity of the medium. $\Omega \subset R^{3}$ is the entire computational domain. We will assume that the material properties are isotropic, but this is not a fundamental restriction.

**Figure 1 Ch1.F1:**
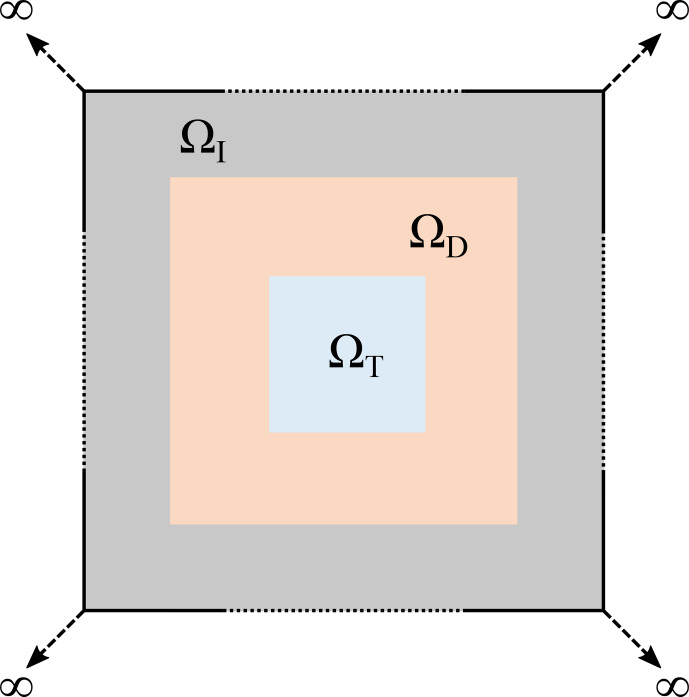
Sketch of the computational domain for the topology optimization of the magnetic lens. The domain in light blue represents the target domain ( $\Omega_{T}$ ), where the magnetic field was focused. The focusing of the magnetic field was achieved by the material interpolation between Cu , and air in the design domain ( $\Omega_{D}$ ) is represented in orange. $\Omega_{D}$ was enclosed by an infinite-element domain ( $\Omega_{I}$ ) in grey. The entire computational domain was $\Omega =\Omega_{T}\cup \Omega_{D}\cup \Omega_{I}$ . Ω was truncated by imposing magnetic insulation boundary conditions on its boundaries ( ∂Ω ).

Figure [Fig Ch1.F1] is a schematic of the rectangular computational domain Ω . The light blue region in the centre represents the target domain ( $\Omega_{T}$ ), where the magnetic field is tailored to be maximized and evenly spread. The region in orange represents the design domain ( $\Omega_{D}$ ), where the material is interpolated between Cu and air to achieve the desired field amplification.

$\Omega_{D}$ is enclosed by an infinite-element domain ( $\Omega_{I}$ ) indicated in grey. $\Omega_{I}$ is stretched rationally by a factor of $10^{3}$ such that the magnetic vector potential ( A ) decays exponentially as a function of distance from the enclosed domain $\Omega_{D}$ . Using a method reported in [Bibr bib1.bibx4], the computation of the wave propagation in $\Omega_{I}$ is fully specified by

6∇2A-ω2μrμ0ϵrϵ0A=0inΩI,7n×∇×A=gon∂ΩD∪∂ΩI,8er×∇×A-jωA=O(r-2)asr→∞,

where n is the normal outward vector on the boundary of $\Omega_{T}$ and $\Omega_{I}$ , g is the tangential magnetic vector potential on $\partial \Omega_{D}\cup \partial \Omega_{I}$ , and $e_{r}$ is the unit vector in the radial direction. Equation (8) represents the exponential decay of the magnetic field. The entire computational domain is truncated by a magnetic insulation boundary condition such that

9 n×A=0on∂Ω.

The topology optimization is achieved based on an adjustable, spatially varying material property. We selected the conductivity of the medium as a function of the spatial coordinates, which interpolates between free space and Cu . To find the values of the conductivity in $\Omega_{D}$ , a design variable ( γ ) was introduced such that γ∈[0,1] , where zero represents free space and unity represents Cu . The variable γ is filtered using a Helmholtz filter equation [Bibr bib1.bibx16]. Based on the finite-element mesh size used for the computation, the radius filter was set to twice the mesh size to avoid ambiguity during the decision phase of material interpolation. The filter equations are

10-r2∇⋅∇γf+γf=γinΩD,11∇⋅γf=0on∂ΩD,

where r is the radius filter, which sets the minimum feature size of the Cu , and $\gamma_{f}$ is the filtered design variable. As γ can have any values between zero and one, it is important to find a solution such that it converges to either of these values. Therefore, in order to reduce intermediate greyscale values so as to achieve a high-contrast material distribution, $\gamma_{f}$ was projected using a hyperbolic tangent function [Bibr bib1.bibx10]:

12 $$\gamma_{p}=\frac{\mathrm{tanh}\;\left(\beta \xi \right)+\mathrm{tanh}\;\left(\beta \left(\gamma_{f}-\xi \right)\right)}{\mathrm{tanh}\;\left(\beta \xi \right)+\mathrm{tanh}\;\left(\beta \left(1-\xi \right)\right)},$$

where β is the projection slope, ξ∈[0,1] is the projection point, and $\gamma_{p}$ is the calculated projected design variable. From Fig. [Fig Ch1.F2]a, it can be observed that γ can be continuously varied between a linear interpolation and a unit step function based on the value of β . To achieve a robust algorithm, β was incremented after a fixed number of iterations. After solving for $\gamma_{p}$ , it is used in the conductivity function to find the density distribution of Cu in $\Omega_{D}$ , which is realized by a combination of logarithmic and power-law expressions [Bibr bib1.bibx7]:

13 $$\sigma \left(\gamma_{p}\right)=10^{\mathrm{log}\sigma_{m}-\left(\mathrm{log}\sigma_{m}-\mathrm{log}\sigma_{\mathrm{air}}\right)\left(1-\gamma_{p}^{p}\right)/\left(1+\gamma_{p}^{p}\right)}\;\;\text{in}\;\;\Omega_{D},$$

where $\sigma_{m}$ is the conductivity of Cu , $\sigma_{\mathrm{air}}$ is the conductivity of the air, and p is the penalization factor. By changing the value of p , the range of conductivity values corresponding to the range of $\gamma_{p}$ could be altered as shown in Fig. S5 in the Supplement. For lower values of p , the design obtained will contain greyscale conductivity values (i.e. for $\gamma_{p}$ besides 0 or 1) that will not have any physical meaning in the real-world environment. To reduce the greyscale it was necessary to increase p . The maximum value up to which it could be increased was limited, as for very high values, the material would be assigned as Cu even when $\gamma_{p}$ is close to zero (i.e. $\gamma_{p}\approx 0$ ). Through trial and error, the optimum value of p was found to be 3. As can be seen from Fig. [Fig Ch1.F2]b, for p=3 , the conductivity varies between metallic values for $\gamma_{p}$ close to 1 and tends to that of free space for values of $\gamma_{p}$ close to 0 .

**Figure 2 Ch1.F2:**
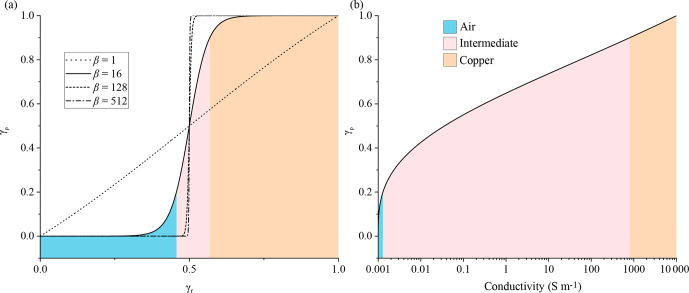
**(a)** Relationship between $\gamma_{f}$ and $\gamma_{p}$ for different values of β . **(b)** Conductivity values as a function of the projected design variable ( $\gamma_{p}$ ) on a logarithmic scale calculated from Eq. (13). As β increases, the intermediate contour in **(a)** decreases, forcing $\gamma_{p}$ to converge to either air or Cu . In **(b)** the air and Cu contours represent the range of conductivity values it can have. With increasing β , the contour area for air and copper decreases, forcing it to have a unique value.

After defining the wave equation and the material distribution equations, control equations were defined to meet the requirements of the MR experiments, i.e. to have a uniform $B_{1}$ distribution along with its enhancement. From the equation of the flip angle

14 $$\alpha =\frac{\gamma_{r}B_{1}\tau }{2\pi },$$

where $\gamma_{r}$ is the gyromagnetic ratio in $\mathrm{MHz}\;T^{-1}$ ; if $B_{1}$ is not uniform, the net magnetization will tip by different angles at different voxel positions, which needs to be minimized, as this reduces the total signal generated. The uniformity of the magnetic field distribution (without affecting the enhancement) was controlled by two equations,

15∫ΩT∇×A∇×Aref-12dΩT,16∫Lξ∇×A∇×Arefdζ≥1forζ∈(x,z),

where $A_{\mathrm{ref}}$ is the large reference magnetic vector potential calculated from Eq. (2) for the reference magnetic field and L ${}_{\xi }$ is one or more diagonal lines in $\Omega_{T}$ . Equation (15) (uniformity control equation) is an error function of the computed magnetic field and the reference magnetic field, and Eq. (16) (field amplification equation) is an inequality function which leads to the computed field to evolve towards the reference field, hence its enhancement. Equation (16) provides computational freedom to find the Cu design while satisfying its condition. At the same time, Eq. (15) is used as an objective function to ensure that the Cu distribution minimizes it. The total area that Cu could occupy in $\Omega_{D}$ was controlled by

17 $$\int_{\Omega_{D}}(\gamma_{p})d\Omega_{D}\leq 0.5,$$

which is a 50 % proportion of $\Omega_{D}$ .

From the above discussion, the optimization problem formulated can be said to be (oxymoronically) a minimization–maximization process, where the minimization of the objective function J leads to a maximization of the magnetic field while maintaining its uniformity. To summarize, the goal of the optimization was to find

18 $$\gamma \in [0,1]\;\;\text{to minimize}\;\;J=\int_{\Omega_{T}}{\left(\frac{\nabla \times A}{\nabla \times A_{\mathrm{ref}}}-1\right)}^{2}d\Omega_{T}$$

subject to

19∇2A-ωμrμ0ϵrϵ02A=-μrμ0σγp∇(ϕ),inΩ,20∇⋅∇(ϕ)=0,inΩ,21-r2∇⋅∇γf+γf=γ,inΩD,22∇⋅γf=0,on∂ΩD,23∫Lζ∇×A∇×Arefdζ≥1,ζ∈(x,z),24∫ΩD(γp)dΩD≤0.5,

where Eqs. (18)–(24) are the field uniformity control equation, the wave propagation equation, the divergence-free condition, the Helmholtz filter equation, the design variable divergence-free condition, the field enhancement equation, the field enhancement equation, and the material control equation, respectively.

The material properties applied to the different domains are represented in Table [Table Ch1.T1]. The implementation of the algorithm, and the results obtained, are discussed in the next section.

**Table 1 Ch1.T1:** Material properties assigned to the different domains for the computation of the optimized geometrical configurations.

Domain	$\mu_{r}$	$\epsilon_{r}$	σ
$\Omega_{T}$	1	80	0
$\Omega_{D}$	1	1	$\sigma (\gamma_{p})$
$\Omega_{I}$	1	1	0

## 3 Numerical implementation

The optimization computations were carried out in the commercial software COMSOL MULTIPHYSICS (V5.4) using its AC/DC and Optimization modules. The simulations were computed using an Intel(R) Xeon(R) Silver 4210 CPU with a processing speed of 2.2 GHz and a RAM size of 64 GB on a 64-bit Windows 10 operating system.

The computational domains were meshed with linear elements to exploit the efficiency of the linear discretization of the magnetic vector potential. This reduced the total computational cost of the simulations. The simulations were formulated such that they follow an iterative procedure as represented by the flowchart in Fig. [Fig Ch1.F3]a. The steps followed sequentially were these.

i. The material properties were initialized in different domains. $\mu_{r}$ and $\epsilon_{r}$ were set to unity for the free space and the conductive material. In $\Omega_{T}$ , where the sample would be placed, $\epsilon_{r}$ was set to 80 and $\mu_{r}$ to 1 to imitate the electromagnetic behaviour of the water. σ was set to zero in all of the domains except in $\Omega_{D}$ , where it was defined as a function of $\gamma_{p}$ described by Eq. (13).
ii. The initial value of the design variable value was set to be 0.5. It was filtered using Eqs. (10) and (11). The filtered design variable was then transformed into a projected design variable using a hyperbolic function described in Eq. (12). The projection point ξ was fixed at 0.5 and the projection slope β initialized at 1, doubled from its previous value after every 30 iterations. With the increasing value of β , Eq. (12) transforms from a linear to a unit step function. The step size for updating β was coarser as the transformation is a rather slow process, and a gradual progression of β would have resulted in $\gamma_{f}$ being projected by a similar function. This would have increased the iteration steps without improving the optimization procedure.
iii. The wave equation was solved using the initial value of the design variable, after which it was updated using the method of moving asymptotes (MMA) [Bibr bib1.bibx20].
iv. The tolerance for the objective function error was set to be a very small number to allow the conditions of Eq. (16) to be met before the premature termination of the simulation. If the constraints defined by Eqs. (16) and (17) were not satisfied until β reaches 1024, the computation terminated automatically.

The optimization was performed for two frequencies, i.e. at 45 and 500 MHz. The results and more detailed setup for these cases are discussed in the following sub-sections.

**Figure 3 Ch1.F3:**
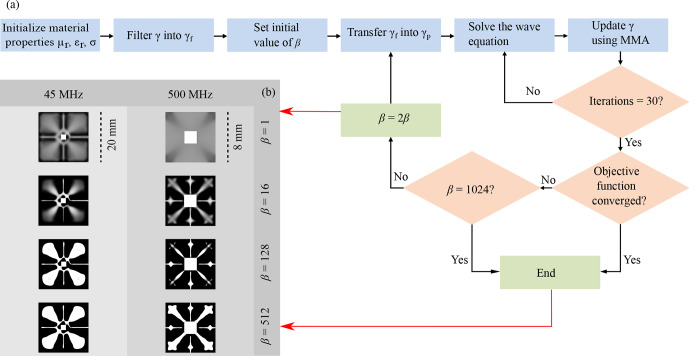
**(a)** Flowchart representing the workflow followed to obtain the material distribution. **(b)** Intermediate results obtained during the optimization process for different values of frequencies, and projection slope ( β ). The material distribution was plotted on a greyscale such that 0 (white) represents air and 1 (black) represents Cu . The final optimization result was obtained at β=512 for the designed frequencies.

### 3.1 Optimization of the magnetic lens at 45 MHz

In an MR experiment, the net magnetization of the sample, which is aligned along the $B_{0}$ field (without loss of generality, the z axis), is flipped by an orthogonal $B_{1}$ field (without loss of generality, the x axis). Thus, the MR coils are designed to produce a unidirectional $B_{1}$ field orthogonal to the $B_{0}$ field. Taking advantage of this condition, we reduced the computational domain to a 2D geometry. A background magnetic field oscillating at a frequency of 45 MHz along an out-of-plane vector was defined to imitate the behaviour of the radio frequency magnetic field generated by an MR coil.

The background magnetic vector potential becomes $A=\left(A(z)\mathrm{exp}(i\omega t),0,0\right)$ , where the magnitude of A(z) was set to $10^{-3}$ Wb m ${}^{-1}$ , which corresponds to a $B_{1}$ of $10^{-3}$ T.

The dimension of the entire computational domain was 24×24 mm ${}^{2}$ . It was limited by the size of the excitation coil which could fit inside the bore of a 1 T preclinical MRI machine (Bruker ICON). The sample was positioned in a 2×2 mm ${}^{2}$ area in $\Omega_{T}$ , surrounded by $\Omega_{D}$ where the material interpolation takes place. $\Omega_{D}$ was enclosed by a 2 mm thick $\Omega_{I}$ .

Figure [Fig Ch1.F3]b shows the evolution of the topology optimization for intermediate values of β . The material distribution obtained was plotted on an inverse greyscale ∈[0,1] , where black corresponding to 1 represents the conductive material and white corresponding to 0 represents the free space. The OL design obtained at the final step, at β=512 , was used for the subsequent post-processing step.

### 3.2 Optimization of the magnetic lens at 500 MHz

A magnetic lens was also designed for an 11.7 T magnet (Bruker AVANCE (III) spectrometer), which corresponds to a Larmor frequency of 500 MHz for ${}^{1}H$ .

The background field was set as defined before in Sect. [Sec Ch1.S3.SS1]. The computational domain in this case was restricted by the size of a commercially available 10 mm saddle coil, which was used for the verification experiments. The entire computational domain was 12×12 ${\mathrm{mm}}^{2}$ . The $\Omega_{D}$ was truncated by a 2 mm thick $\Omega_{I}$ to reduce it to a dimension of 8×8 ${\mathrm{mm}}^{2}$ . The sample was placed in a 2×2 ${\mathrm{mm}}^{2}$ area in $\Omega_{T}$ .

Figure [Fig Ch1.F3]b shows the intermediate results of the topology optimization at every 30th iteration. The material distribution obtained was plotted on a reverse greyscale as defined in Sect. [Sec Ch1.S3.SS1].

## 4 Post-processing

After the designs of OLs were obtained, they were characterized and compared with a wired LL similar to that discussed by [Bibr bib1.bibx15] and [Bibr bib1.bibx13].

To characterize the magnetic field distribution and its enhancement, a second simulation environment was set up where the background field was replaced by the magnetic field produced by a realistic coil geometry. The boundaries of the OLs were truncated by an IBC, and electromagnetic properties of Cu were assigned to it.

An OL is designed to enhance a unidirectional magnetic field. It focuses the magnetic field for coils exhibiting this property (Fig. S2), but for characterization, only a solenoidal coil type was used since the OL properties would be similar to the other coils. Figure [Fig Ch1.F4]c and d show the amplification profile of the magnetic field by the OLs in a solenoidal coil type arrangement. The coils had an outer radius of 23 mm (for 45 MHz) and 6 mm (for 500 MHz), respectively. The two conductive rings were separated by a distance of 3.2 mm, based on the thickness of the PCB used for the verification measurements. Figure [Fig Ch1.F4]b and e represent the current induced in the OLs. In Fig. [Fig Ch1.F4]b, the design obtained for the OL was asymmetric. At lower frequencies the strength of inductive coupling is weak; therefore, the current flow produced by the Cu distribution results in the desired magnification, while the protrusions maintain the uniformity of the field. As the magnitudes of the electric and magnetic fields scale linearly with the frequency, at higher frequencies, stronger inductive coupling ensures the enhancement. This forces the algorithm to produce a more symmetric Cu distribution to reduce the unfeasibilities of the control equations.

**Figure 4 Ch1.F4:**
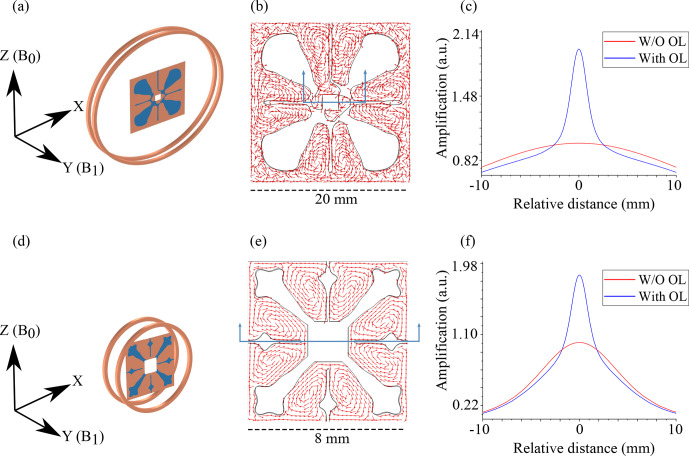
The setup used for characterizing the OL: **(a)** 45 MHz and **(d)** 500 MHz. The OLs were placed at the centre of two conductive rings such that the magnetic field ( $B_{1}$ ) produced by the coil is normal to its plane. $B_{0}$ represents the direction of the static magnetic field to visualize the orientation of the arrangement when placed in the MR machine. **(b, e)** show the currents induced in the OL. **(b)** From the current flow, it can be interpreted how the magnetic flux would be concentrated at the $\Omega_{T}$ , while the current flowing on copper protrusions ensures the uniformity of the magnetic field. **(e)** At 500 MHz due to stronger inductive coupling, the amplification produced is high; therefore, the optimization results in a symmetric material distribution in order to maintain the uniformity while at the same time ensuring a desired current flow for the field amplification. **(c, f)** Amplification produced by the OLs, plotted along the direction of $B_{1}$ . The distance represents relative values from the centre point of the OLs.

To compare the OLs with the LLs, geometries similar to those as shown in the inset of Fig. [Fig Ch1.F5] were used. The total magnetic flux in the target domain without any lens and with the lens was calculated using the equation

25 $$B_{\mathrm{total}}=\frac{\int_{\Omega_{T}}B_{1}d\Omega_{T}}{\int_{\Omega_{T}}1d\Omega_{T}}.$$

For the 45 MHz arrangement, without any lens, it was 47.94 µT , and after positioning the OL in the coil, the total magnetic flux calculated was 105.31 µT , which resulted in a field magnification of 2.2.

The OL was then replaced with an LL. The LL had an outer diameter of 19 mm, with an outer to inner diameter ratio of 5.59. The total magnetic flux calculated for this arrangement was 139.55 µT , which resulted in the field magnification of 2.9.

The LL was found to achieve better enhancement of the field compared to the OL. However, the field distribution for the OL was more uniform. The field uniformity was calculated as the deviation from the $B_{1}$ at the centre of the lens in the test region $\Omega_{T}$ :

26 $${\left.B_{\mathrm{deviation}}\right|}_{\Omega_{T}}=\left(\frac{B_{\mathrm{centre}}-B_{1}}{B_{\mathrm{centre}}}\right)\times 100\;\%\;\;\;\text{in}\;\;\;\Omega_{T}.$$

Without any lens, the deviation calculated was 0.3 %. For the OL, it was 33.72 %, and for the LL it was 39.93 %. Similarly, the deviations along the central lines were calculated as ${\left.B_{\mathrm{deviation}}\right|}_{L_{z}}$ and ${\left.B_{\mathrm{deviation}}\right|}_{L_{x}}$ . The maximum deviation for the OL was calculated to be 20.8 % along the z axis, and for the LL it was 23.35 % along the x axis (see Table [Table Ch1.T2]).

**Table 2 Ch1.T2:** Comparison summary between the OL and the LL. For the LL, the values in brackets represent the ratio of the outer to the inner diameter.

Lens	Frequency	Amplification	Variation in $\Omega_{T}$	Variation along $L_{z}$ and $L_{x}$
LL(5.59)	45 MHz	2.9	39.93 %	23.35 %
Optimized lens	45 MHz	2.2	33.72 %	20.8 %
LL(2.24)	500 MHz	1.79	33.6 %	19 %
Optimized lens	500 MHz	2	33.4 %	17 %

By increasing the frequency of operation for this particular arrangement to 500 MHz, the LL produced a magnification of 3, which is slightly higher than at 45 MHz, and the OL produced a magnification of 3.9. As can be seen from the design due to the asymmetric material distribution, with the OL the field distribution of the magnetic field was less central. If the region of interest is reduced such that the variation lies below 10 %, the OL at higher frequencies can still be used; therefore, depending on the application, one can also use the magnetic lens designed for 45 MHz at 500 MHz to get a higher amplification if maintaining uniformity is not a concern or a smaller sample volume can be used. In order to get a uniform field distribution following the same protocol for the optimization as described in Sect. [Sec Ch1.S3.SS1] and designing the magnetic lens at the same dimensional limits for 500 MHz, the eccentricity issue of the previous design was fixed while the magnification obtained was 2. The OL obtained for such an arrangement is shown in Fig. S1.

The magnification of the magnetic field produced by the LL depends directly on the ratio of the outer to inner diameter. For 1.05 T (Bruker ICON) measurements, the ratio of the coil size to the sample size was large enough to have a higher amplification by the LL. If we reduce the size of the coil by keeping the sample dimensions the same, as was the case for 11.7 T (Bruker AVANCE (III)) measurements, where a commercially available Bruker's 10 mm saddle coil was used, this leads to a reduction in amplification produced by the LL. The OL designed in Sect. [Sec Ch1.S3.SS2] was able to produce a better amplification while maintaining the uniformity.

To verify this, the OL and the LL were analysed in a solenoidal coil arrangement. The outer radius of the coil was 6 mm. The two rings were separated by a distance of 3.2 mm. The coils were excited with an alternating current oscillating at a frequency of 500 MHz.

The total magnetic flux in the volume of the sample calculated using Eq. (25) without any lens was 179.42 µT , and after placing the OL, the total flux calculated was 365.65 µT which resulted in an amplification factor of 2.04.

By replacing the OL with an LL whose outer diameter was 7.6 mm and outer to inner diameter ratio 2.24, the total magnetic flux was 322 µT , which resulted in a total amplification factor of 1.79.

Therefore, with the reduction in the outer to inner diameter ratio, the total magnification produced by the LL is also reduced. However, the OL was still able to maintain the defined amplification. The maximum variation along the central lines calculated from Eq. (26) for the LL was 19 % along the x axis, and for the OL it was 17 % along the z axis. However, the total magnetic field variation for both types was comparable at 33 % (see Table [Table Ch1.T2]). Without any magnetic lens in the setup, it was 2.6 %.

To summarize the discussion, the LL was found to have a higher magnification, but the field distribution was less uniform. When the ratio of the outer to the inner diameter for the LL is reduced, it produces a lower magnification compared to the optimized magnetic lens albeit the field uniformity of these devices was similar. Table [Table Ch1.T2] summarizes the comparison result and Fig. [Fig Ch1.F5] shows the amplification profiles for different lenses.

**Figure 5 Ch1.F5:**
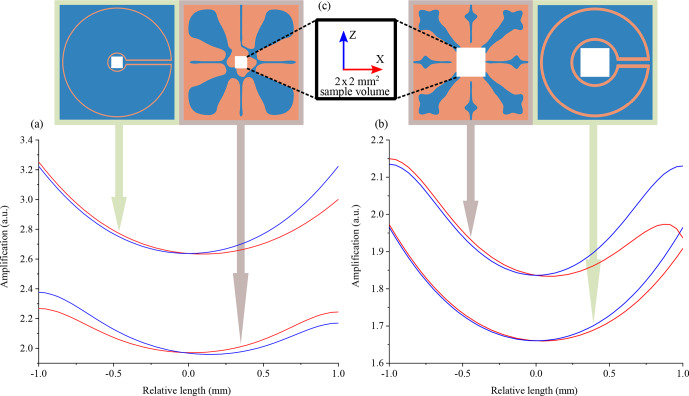
**(a, b)** Amplification profile comparison of the OL with the LL in the x – z plane in $\Omega_{T}$ . The horizontal axis represents the relative distance from the centre point of $\Omega_{T}$ , in the x and z directions. **(a)** The frequency of operation was 45 MHz. The LL's outer diameter was 19 mm, and the ratio of the outer to the inner diameter was 5.59. **(b)** The frequency of operation was 500 MHz. The LL's outer diameter was 7.6 mm, and the ratio of the outer to the inner diameter was 2.24. **(c)** The sample region in the centre of the Lenz lenses is always a 2×2 mm square.

From the above discussion a question arises: why not set a reference field to achieve an amplification of 5 times rather than a mere factor of 2? The reason a reference field was not set higher is that this leads to the material not being properly distributed and we get undefined conductivity values, i.e. at greyscale values besides 0 or 1.

## 5 Fabrication and experimental verification

After processing the designs from the simulations, they were fabricated and verified with NMR experiments using distilled water as a test sample.

### 5.1 Fabrication

The masks for the designs were printed on butter paper using an HP Laserjet Enterprise P3015 dn printer.

Using the mask for UV lithographic patterning, the designs were copied onto a positive photosensitized copper board with FR4 laminate with a PCB thickness of 1.6 mm and a Cu thickness of 35 µm , obtained from an external supplier (C.I.F., France).

The board was etched in a sodium persulfate solution ( ${\mathrm{Na}}_{2}S_{2}O_{8}$ ). The etching solution was prepared such that for every 1 L of DI water, 1 g of ${\mathrm{Na}}_{2}S_{2}O_{8}$ was dissolved in it. The etching solution was placed in a bubble etch tank (PA104 (Mega Electronics), with etching time varying from 25 to 60 min depending on the age of the etching solution.

For NMR characterization, a 0.5 µL sample was prepared in a capillary with an inner diameter of 0.8 mm. The sample occupied a length of 1 mm of the tube.

### 5.2 NMR experimental protocol

The low-frequency magnetic resonance measurements were acquired by placing the OL in a solenoidal coil, whereas for the high-frequency measurements a saddle coil was used, as shown in Fig. [Fig Ch1.F6]a and d.

**Figure 6 Ch1.F6:**
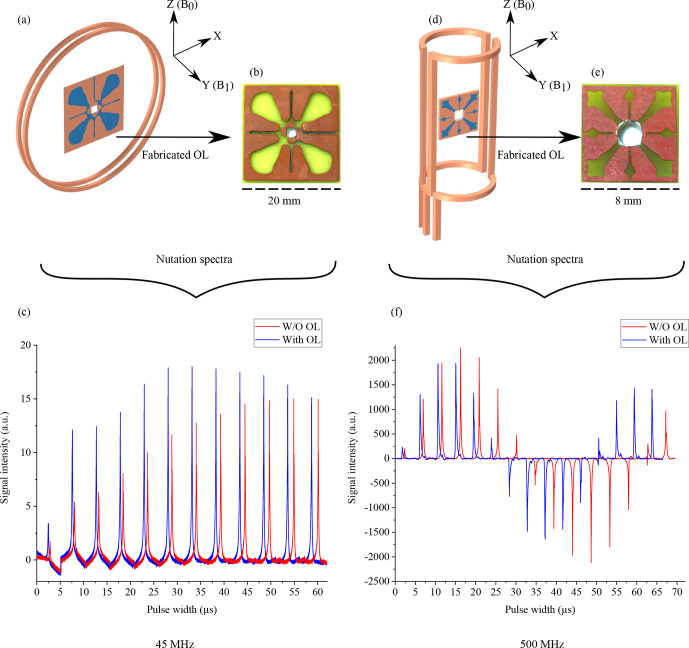
**(a, d)** The coil type and OL arrangement used for the NMR measurement at 45 MHz. The axis represents the orientation of the device in the NMR measurement apparatus. **(b, e)** Fabricated OLs on the PCBs. **(c, d)** Fourier transformation of the measured nutation spectra with and without the OLs.

Both OLs featured self-resonance frequencies in the GHz range, as shown in Fig. S3; therefore, combining them with an inductively coupled, tuned, and matched coil did not affect the overall resonance frequency, nor was the quality factor significantly degraded (see Fig. S4). The matching conditions were affected and could be corrected by the probe coil's variable capacitor.

For the NMR experiments, all acquisitions were done in single shot without any averaging. The coils were positioned at the iso-centre of the $B_{0}$ field. The measurements were initialized using the coil without any lens to adjust the power and the acquisition time. After initialization, a nutation spectrum was acquired to determine the 90 ${}^{\circ }$ flip angle.

Next, the OL was introduced in the coil. The shimming profile had to be re-adjusted in order to obtain a similar spectral line width for both experiments. With the same volume of the sample, acquisition time, and power to the coil, a second nutation spectrum experiment was acquired to determine the change in 90 ${}^{\circ }$ flip angle and to characterize the $B_{1}$ uniformity of the OL.

The relative intensities of the two arrangements were determined from the areas under the spectrum for a 90 ${}^{\circ }$ flip angle to show signal enhancement. The noise values were calculated as the deviation of the signal at the baseline of the spectrum, taken in a peak-free region. The SNR was calculated as the ratio of the area under the peak signal divided by the noise. Table [Table Ch1.T3] summarizes the relative SNR values and $B_{1}$ field enhancement calculated by Eq. (14).

**Table 3 Ch1.T3:** Values calculated from the nutation spectra of water at Larmor frequencies of 45 and 500 MHz, respectively. The values represent the ratio of the SNR, $B_{1}$ enhancement, and the pulse duration to produce the 90 ${}^{\circ }$ flip angle. The comparison was made with and without the OL.

Frequency	Relative	$B_{1}$	90 ${}^{\circ }$ pulse
in MHz	SNR	enhancement	duration in
			µs with OL
			(w/o OL)
45	1.56	1.66	33.15 (54.9)
500	1.19	1.3	12.5 (16.23)

The measurements at 45 MHz proved difficult, mainly due to the large magnetic field drift experienced for the ICON system, exacerbated by the lack of a lock channel on the device. The strategy was to acquire nutation spectra as quickly as possible, i.e. at a rather large step size, to estimate the 90 ${}^{\circ }$ flip angle. Nevertheless, through experimental verification, we were able to asymptotically determine the magnetic field amplification and hence the improvement of the SNR using an OL geometry.

## 6 Conclusions

It is hardly a surprise that the quest for more signal-to-noise from an existing NMR detector arrangement without changing other conditions like sample volume, radiofrequency power applied, coil geometry, etc., is a matter of numerical optimization of a passive element which can improve the filling factor of the coil. Topology optimization offers a feasible pathway with which to reach optimal designs that goes beyond mere intuition, and we could show, using a commercial finite-element tool, that it is possible to find practical Lenz lens arrangements that, when implemented, achieve their set goals. The topologies found form a compromise between signal enhancement and field uniformity. Of course, it would have been possible to extend the Pareto front (set of all Pareto-efficient solutions, [Bibr bib1.bibx12]) to include additional goals, such as maintaining a good susceptibility shift profile. Our experience is, however, that this leads to difficulties, mainly because the optimization problem becomes over-constrained and hence no longer evolves towards useful design modifications.

We only discussed the use of optimization to enhance the magnetic field of an MR coil, but of course the methodology could be further extended to design a self-resonant structure in order to avoid the tedious task of matching to the characteristic impedance of the coaxial cable (usually 50 Ω ) and tuning to the Larmor frequency. A properly matched and tuned detector improves the signal transmitted during the excitation phase and the signal received during the signal acquisition phase. In this regard, the designs found reconfirmed one useful aspect of Lenz lens arrangements, namely, that they do not modify the tuning of the outer driver coil, merely its matching condition (the depth of the absorption dip in the $S_{11}$ curve), and suitable reflection conditions are easily found.

An important aspect is the ability to achieve manufacturable designs. For the case where a design is essentially a 2D metal patch on a dielectric sheet, printed circuit boards are an inexpensive route towards implementation, easy to manufacture, and lead to satisfying results for arbitrary embedded topologies. However, for the case of 3D topologies, i.e. to find the material distribution in 3D space, the situation is quite different, and not all geometries found will be manufacturable. For example, to minimize eddy current losses, by breaking the continuity of the induced current (due to varying magnetic fields) away from the sample region, designs tend to evolve towards tiny disconnected islands of metal, arranged in a dielectric background or metal patch suspended in air. This would require very advanced 3D printing, and in some cases the designs might not even be pragmatic. We did not pursue such designs in this contribution, but the message should be clear. Optimization must include manufacturing constraints in order to achieve feasible designs.

Beyond manufacturability, a design must be practicable in use, which further limits the design freedom, because the sample must be provided with a convenient way in and way out of the sensitive volume of the detector. We found no problems with 2D designs, but 3D designs posed a challenge, resulting in designs that left too little space.

The general conclusion is that all aspects of a design must be mathematically expressible as a goal function in order to be considered, but, as more terms are added to the optimization goal, the numerical convergence process slows down, eventually reaching a standstill.

Because computational electromagnetics is scale invariant, the topology optimization methodology is applicable to resonator arrangements beyond the range of typical nuclear magnetic resonance frequencies, applications such as the design of magnetic or electric resonators used in electron paramagnetic resonance (EPR), the optimization of individual wavepath components including capacitors and strip lines, and even for wireless energy transfer.

## Supplement

10.5194/mr-1-225-2020-supplement The supplement related to this article is available online at: https://doi.org/10.5194/mr-1-225-2020-supplement.

## Data Availability

Simulation and measurement data used for the figures are available at https://doi.org/10.5445/IR/1000124281 (Wadhwa et al., 2020).
