# Supplementary material for: Topologically optimized magnetic lens for magnetic resonance applications
Source: Magn Reson (Gott). 2020 Oct 12;1(2):225–36. doi: 10.5194/mr-1-225-2020 (PMC10500734; doi:10.5194/mr-1-225-2020)
Supplement: The supplement related to this article is available online at: https://doi.org/10.5194/mr-1-225-2020-supplement. [file mr-1-225-supplement.pdf]

Supplement of Magn. Reson., 1, 225–236, 2020
https://doi.org/10.5194/mr-1-225-2020-supplement
© Author(s) 2020. This work is distributed under
the Creative Commons Attribution 4.0 License.

Open Access
MAGNETIC RESONANCE

Supplement of

Topologically optimized magnetic lens for magnetic resonance applications

Sagar Wadhwa et al.

Correspondence to: Jan G. Korvink (jan.korvink@kit.edu)

The copyright of individual parts of the supplement might differ from the CC BY 4.0 License.

1 OL Optimization

The optimized Lenz lens was designed to operated at different frequencies for the dimensional limits specified in the paper. The 8 mm square lens was designed for a frequency of operation at 45 MHz, and the 20 mm square lens for operation at 500 MHz. As discussed in the paper Section 4, low frequency designs lead to asymmetric structures, and high frequency design leads to highly symmetric structures. Figure 1 shows the material distribution obtained, where white (0) represents air, and black (1) represents *Cu*.

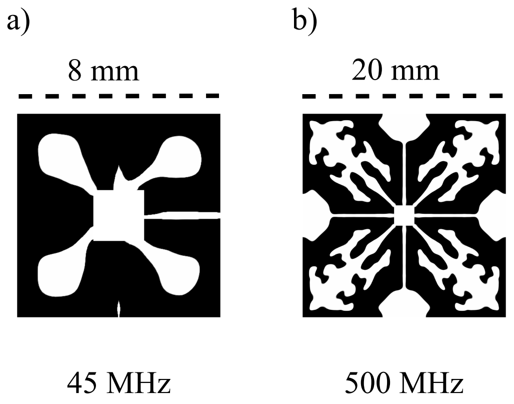

Figure 1. (a) Optimized 8 mm Lenz lens design obtained for operation at 45 MHz. (b) Optimized 20 mm Lenz lens design obtained for operation at 500 MHz. The material distribution obtained from the simulation was gray scale, and was sharpened to a binary black & white distribution using a penalisation method. Here, black represents conductive copper. The imperfections in layout are due to irregularities in the finite element mesh, and disappear for sufficiently fine discretisations. Geometry lies in the y-z plane, perpendicular to the $\boldsymbol{B}_1$ field which lies along the x-axis.

2 Amplification Profile for Different Coil Types

Since each lens was optimized for a unidirectional $\boldsymbol{B}_1$-field ($\boldsymbol{B}_1 \parallel \boldsymbol{x}$), the optimized Lenz lens can be used with any coils exhibiting this property. Figure 2 shows the amplification profile plotted for the optimized Lenz lens optimized for 45 MHz, and dimensional limit of 23 mm; Figure 2(a), shows amplification profile the profile for a saddle coil, and Figure 2(b) shows the amplification profile for a 3 turn saddle coil. The coils had a radius of 23 mm

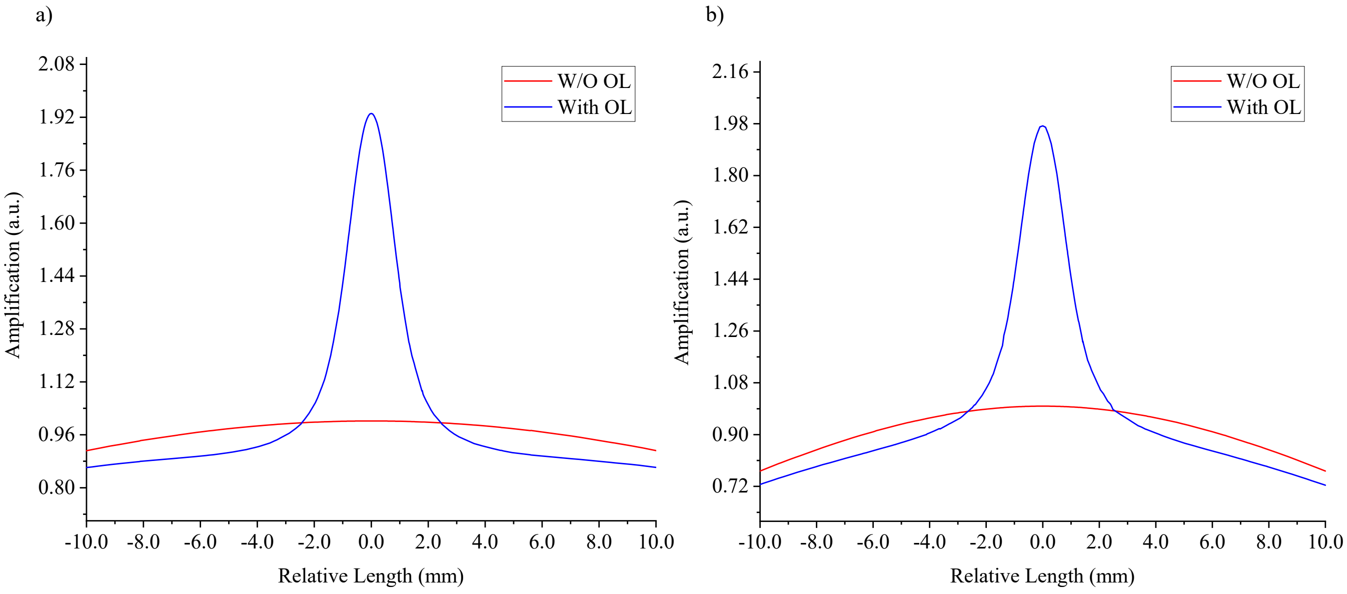

Figure 2. Amplification profile for an optimized 20 mm Lenz lens operating at 45 MHz. Different coils were used for the excitation: (a) saddle coil; (b) solenoidal coil with 3 turns. The Lenz lens was placed at the centre of each coil such that $\boldsymbol{B}_1$ remained parallel to the original field of the coil. The relative field amplification profile relative to the coil centroid field (without a Lenz lens) is plotted as a function of the $\boldsymbol{x}$-axis coordinate $\parallel \boldsymbol{B}_1$.

3 Frequency Response of Magnetic Lenses

Lenz lenses are broadband devices up to their resonance frequency. Figure 3 shows the absorption spectra for different sized Lenz lens. At the resonance frequency, the magnetic field at the centre of the device is amplified, whereas electric fields are primarily formed in regions where metal tracks are in close proximity and, simultaneously, currents are out of phase or differ in amplitude. The absorption spectra were obtained from the simulation, where each lens was positioned inside a rectangular waveguide. An *RF* excitation was applied from one end of the waveguide normal to the lens, and a receiver placed at the other end was used to measure the total power received (the transmission amplitude). From this absorption spectra were evaluated over a frequency sweep. The length of the waveguide was set smaller than the shortest wavelength of excitation, to avoid standing wave conditions. The waveguide was hollow, and electromagnetic properties of free space were used in its cavity.

Since the resonance frequency of 10-20 mm square Lenz lenses are in the range of 2-7 GHz, and hence behave as pure inductors at lower NMR frequencies, introducing such a lens into a tuned and matched coil circuit did not add significant capacitance to the effective resulting impedance. The matching could thus be easily corrected by varying probe's capacitors. Figure 5 shows the change in matching condition before and after the lens is inserted. The S-parameter were measured using a network analyzer (Agilent Technologies E5071C (100 kHz-8.5 GHz)).

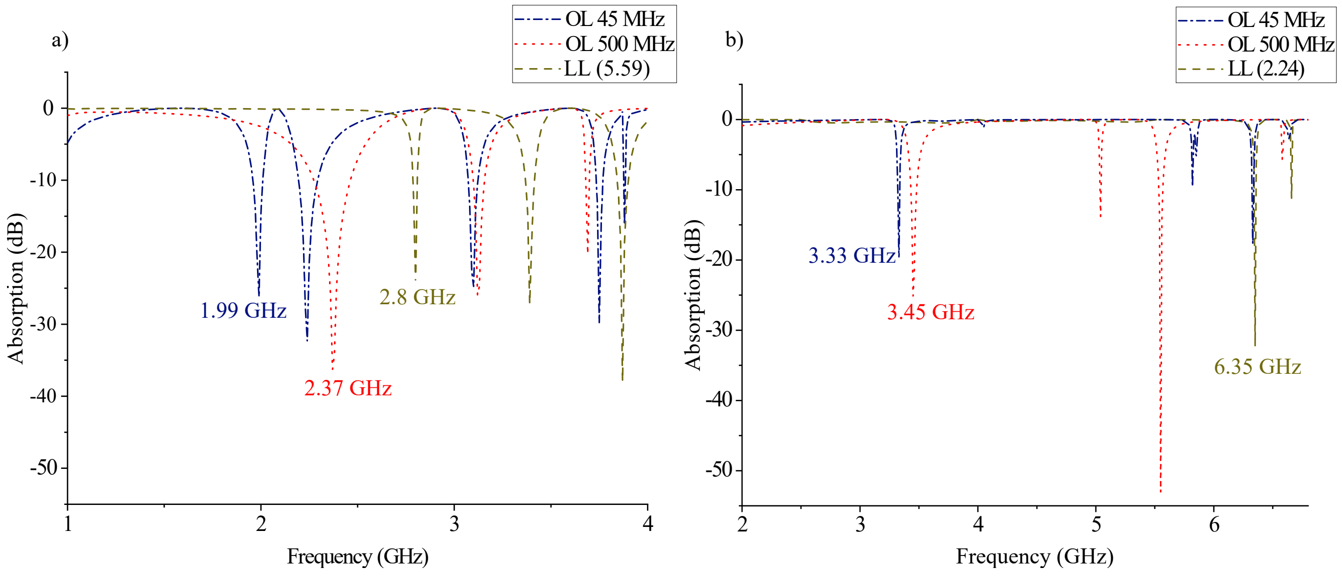

Figure 3. Simulated absorption spectra for Lenz lenses designed for different frequencies and sizes: (a) 20 mm square, and (b) 8 mm square. The dip annotation on each graph represents the fundamental resonance frequency for a particular lens. In the caption, for each *LL*, the value in brackets represents the ratio of inner to outer diameter.

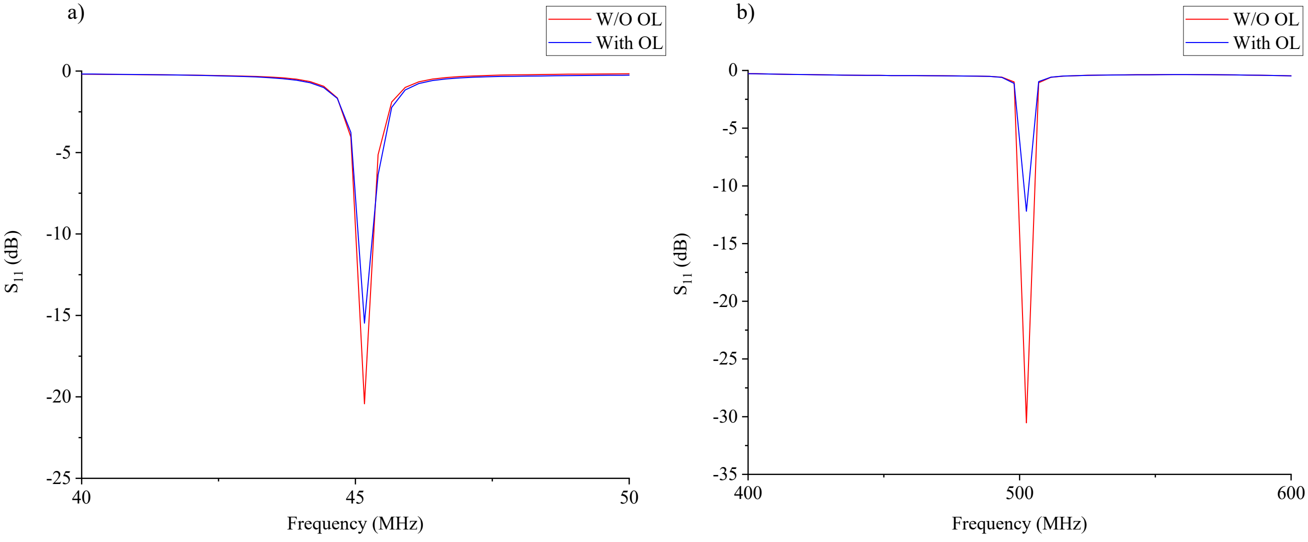

Figure 4. The S_{11} parameters measured with and without each of the two optimized Lenz lenses which were selected for NMR measurement. For each case, the coil was initially matched at 50 ohms, and tuned for either (a) 45 MHz, or (b) 500 MHz. Subsequently, the custom optimized Lenz lens was introduced and, as can be observed from the graph, only the matching conditions were effected (the depth of each dip) whereas the tuning did not change (the frequency position of each dip).

4 Response of the Conductivity Function with respect to the Penalization Factor

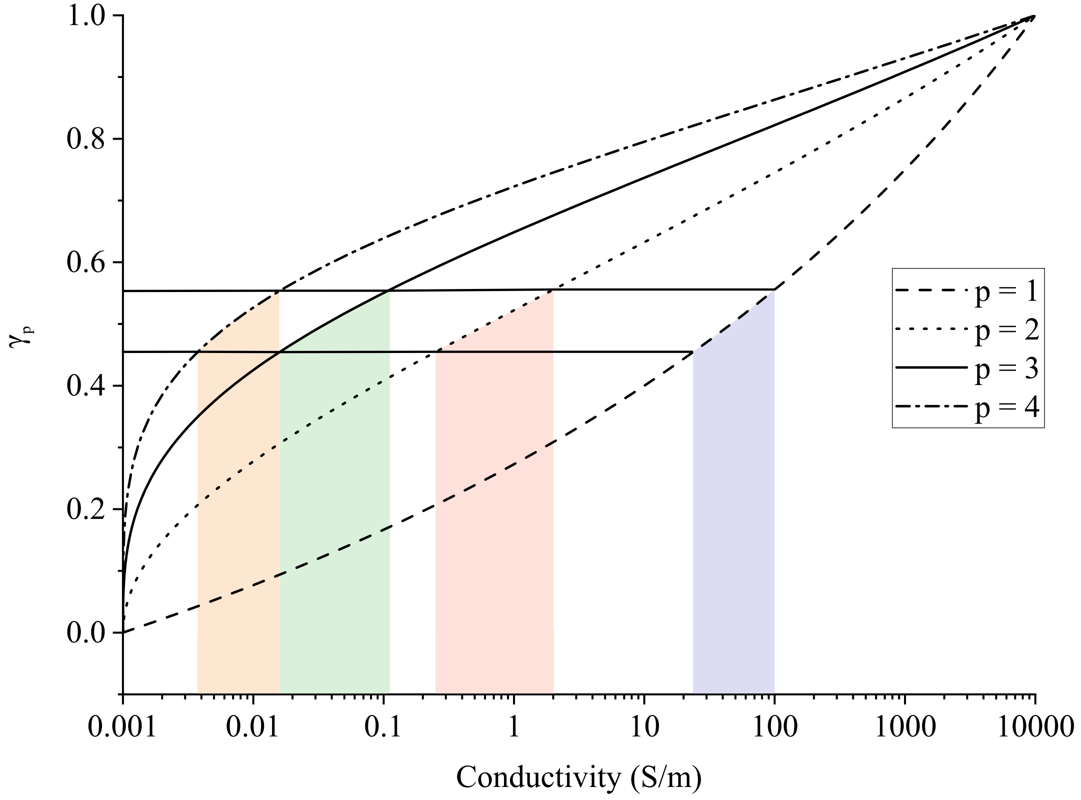

Figure 5. Response of the conductivity function for different values of the penalization factor. The shade regions corresponds to the conductivity values for $\gamma_p \in$ [4.5,5.5].
